# Serum metabolomics analysis in patients with alcohol dependence

**DOI:** 10.3389/fpsyt.2023.1151200

**Published:** 2023-04-17

**Authors:** Yanjie Zhang, Yajun Sun, Qin Miao, Shilong Guo, Qi Wang, Tianyuan Shi, Xinsheng Guo, Shuai Liu, Guiding Cheng, Chuansheng Wang, Ruiling Zhang

**Affiliations:** ^1^Department of Psychiatry, Henan Mental Hospital, The Second Affiliated Hospital of Xinxiang Medical University, Xinxiang, China; ^2^Henan Key Lab of Biological Psychiatry, Xinxiang Medical University, Xinxiang, China; ^3^Department of Scientific Research, The Second Affiliated Hospital of Xinxiang Medical University, Xinxiang, China; ^4^Department of Addiction, The Second Affiliated Hospital of Xinxiang Medical University, Xinxiang, China; ^5^Department of Oncology, The Third Affiliated Hospital of Xinxiang Medical University, Xinxiang, China

**Keywords:** alcohol dependence, liquid chromatography-mass spectrometry (LC–MS), metabolic signal pathways, multivariate statistical analysis, potential biomarkers

## Abstract

**Objective:**

Alcohol dependence (AD) is a chronic recurrent mental disease caused by long-term drinking. It is one of the most prevalent public health problems. However, AD diagnosis lacks objective biomarkers. This study was aimed to shed some light on potential biomarkers of AD patients by investigating the serum metabolomics profiles of AD patients and the controls.

**Methods:**

Liquid chromatography-mass spectrometry (LC–MS) was used to detect the serum metabolites of 29 AD patients (AD) and 28 controls. Six samples were set aside as the validation set (Control: *n* = 3; AD group: *n* = 3), and the remaining were used as the training set (Control: *n* = 26; AD group: *n* = 25). Principal component analysis (PCA) and partial least squares discriminant analysis (PCA-DA) were performed to analyze the training set samples. The metabolic pathways were analyzed using the MetPA database. The signal pathways with pathway impact >0.2, value of *p* <0.05, and FDR < 0.05 were selected. From the screened pathways, the metabolites whose levels changed by at least 3-fold were screened. The metabolites with no numerical overlap in their concentrations in the AD and the control groups were screened out and verified with the validation set.

**Results:**

The serum metabolomic profiles of the control and the AD groups were significantly different. We identified six significantly altered metabolic signal pathways, including protein digestion and absorption; alanine, aspartate, and glutamate metabolism; arginine biosynthesis; linoleic acid metabolism; butanoate metabolism; and GABAergic synapse. In these six signal pathways, the levels of 28 metabolites were found to be significantly altered. Of these, the alterations of 11 metabolites changed by at least 3-fold compared to the control group. Of these 11 metabolites, those with no numerical overlap in their concentrations between the AD and the control groups were GABA, 4-hydroxybutanoic acid, L-glutamic acid, citric acid and L-glutamine.

**Conclusion:**

The metabolite profile of the AD group was significantly different from that of the control group. GABA, 4-hydroxybutanoic acid, L-glutamic acid, citric acid, and L-glutamine could be used as potential diagnostic markers for AD.

## Background

Alcohol is one of the most widely used psychoactive substances. Clinical research shows that both the number of alcohol drinkers and the annual alcohol consumption are increasing (1). The disease burden caused by alcohol consumption is also increasing (2). Alcohol use disorder, is the most prevalent mental disorder in the world, associated with high mortality and disease burden (3). It is characterized by compulsive and uncontrolled drinking, including alcohol abuse and alcohol dependence (AD), which is caused by long-term drinking. AD, also known as alcohol addiction, is manifested in the form of forced alcohol seeking and continuous or regular drinking. Its core symptoms include physical dependence, characterized by increased alcohol tolerance and withdrawal reaction, and psychological dependence, characterized by alcohol craving. AD is the most serious type of alcohol use disorder, with a prevalence rate of 2.6% among people elder than 15 years old in 2016 (4). America has the highest prevalence rate (4.1%), followed by Europe (3.7%) (4) and China (1.3%) (5). At present, AD and its related problems are the third most prevalent global public health problem after cardiovascular diseases and tumors (6). Therefore, it has attracted much attention of people, especially researchers.

The researches on AD mainly focus on genetics (7, 8), epigenetics (9, 10), imaging (11–13), etc. Only a few studies have focused on identifying metabolic markers of AD through metabolomics analyses. However, AD patients may be accompanied by metabolic abnormalities. Many AD patients have irregular lives, and heavy drinking worsens their appetite. Alcohol can only provide energy. It does not contain essential nutrients, such as proteins, required by the body. In addition, AD patients have impaired gastrointestinal and liver functions and absorption obstacles. Thus, the lack of nutrients is a problem for serious alcoholics. These factors can be contributed to metabolic abnormalities in AD patients.

Metabolomics, which is usually based on chromatography and mass spectrometry, have been used to better understand diseases and drug discovery (14–23). LC–MS is a widely used metabolomics technique (24). Principal component analysis (PCA) and partial least squares-discriminant analysis (PLS-DA) are the two most commonly used dimensionality reduction analysis methods for metabolomic data to reduce the complexity of the data (25).

Metabolomics has divided into untargeted and targeted metabolomics based on the research purpose. Untargeted metabolomics has been commonly used in human diseases, such as type 2 diabetes (26), liver disease (27), cancer (28, 29), neurological disorders (30), and drug addiction (31, 32). However, studies on AD-related metabolomics are rare.

In this study, an untargeted LC–MS approach was used to study the serum metabolomics of AD patients and the controls. We analyzed the differential metabolic pathways and metabolites potentially associated with AD. Our findings might provide useful insights into the pathogenesis of AD and associated biomarkers.

## Materials and methods

### Reagents and instruments

Methanol and acetonitrile were obtained from Thermo Fisher Scientific (United States). 2-Chlorophenylalanine and formic acid were purchased from Aladdin (Shanghai, China) and Tokyo Chemical Industry (Shanghai, China), respectively. Ammonium formate was purchased from Sigma Aldrich (USA). The frozen centrifuge with the model of H1850-R used in the experiment comes from Xiangyi (China). The mixer with the model of BE-2600 was from Kylin-Bell (China) and the vacuum concentrator (model 5,305) was from Eppendorf (Germany). The filter membrane (0.22 μm) were obtained from Jinteng company (China). Metabolomics analysis was conducted on Thermo Fisher liquid chromatography in tandem with a Q Exactive HF-X Hybrid Quadrupole-Orbitrap Mass Spectrometer (Thermo Fisher Scientific, United States).

### Study population

**Figure 1:**
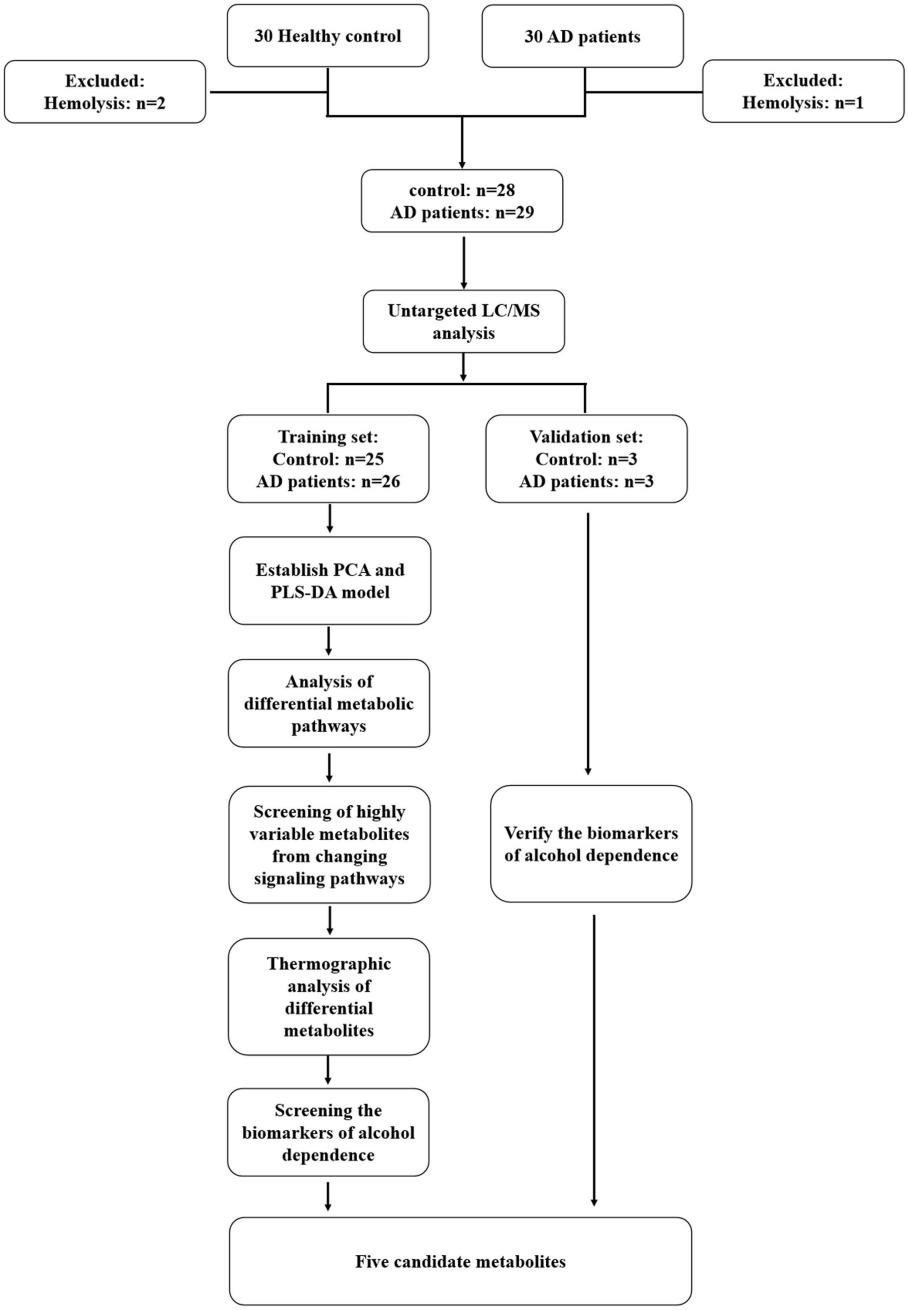
The flowchart of the study protocol.

The AD patients admitted to the Department of Addiction in the Second Affiliated Hospital of Xinxiang Medical University between December 2019 and December 2020 were enrolled in this study. The control serum samples were acquired from healthy normal individuals during the same period. The flowchart of the study protocol is demonstrated in Figure 1.

### Sample preparation for metabolomics analysis

Fasting blood was collected into non-anticoagulant blood collection vessels in the morning and coagulated at 4°C for 8 h. Then, it was centrifuged at 4°C at 3000 rpm for 15 min. The serum was temporarily stored at −80°C before use. Hemolytic samples were excluded. Before the metabolomics analysis, the sample was thawed at 4°C. Each sample with a mount of 100 μL was transferred into 2 mL centrifuge tubes, respectively. Then, 400 μL of precooled methanol (at −20°C) was added to each tube and mixed well. The mixtures were then centrifuged for 10 min at 12000 rpm at 4°C. The supernatants were transferred to another 2 mL centrifuge tube. The samples were vacuum dried, concentrated, and re-dissolved in 150 μL of 80% methanol solution (methanol: water(v/v, 4:1)), respectively. 2-chlorobenzalanine with a final concentration of 4 μg/mL, was added in the above solution as an internal standard to verify instrument stability. The mixture was then filtered with a separate 0.22-μm membrane. Quality control (QC) samples was prepared by mixing all the sample (each sample volume: 20 μL). The remaining of each sample was used for LC–MS.

### Metabolomics analysis by LC–MS

The chromatographic and mass spectrometric conditions used in this study were according to previous studies (33). Briefly, an ACQUITY UPLC® HSS T3 column (150 × 2.1 mm, 1.8 μm diameter, Waters) was used for chromatographic separation. Two microliters of each sample was analyzed after the column equilibrated at a constant temperature of 40°C. Mass spectrometry spectra were sampled with an electrospray ionization source (ESI) operating both in positive-ion (voltage: 3.5 kV) and negative-ion mode (voltage: −2.5 kV). The eluents of 0.1% formic acid in water (A) and 0.1% formic acid in acetonitrile (B) was used for ESI+ mode analysis and 5 mM ammonium formate in water (C) and acetonitrile (D) for ESI-mode analysis. Gradient elution was used as follows: 0 ~ 1 min, 2% B/D; 1 ~ 9 min, 2% ~ 50% B/D; 9 ~ 12 min, 50% ~ 98% B/D; 12 ~ 13.5 min, 98% B/D; 13.5 ~ 14 min, 98% ~ 2% B/D; 14 ~ 20 min, 2% D-positive model (14 ~ 17 min, 2% B-negative model). Flow rate of the eluent is set at 0.25 mL/min. The capillary temperature was set at 325°C. Data-dependent acquisition (DDA) MS/MS experiments were performed with a higher-energy C-trap dissociation (HCD) scan. The normalized collision energy was set at 30 eV. Dynamic exclusion was implemented to remove some unnecessary information in MS/MS spectra.

Xcms format files are used for peak identification, filtration, and alignment, which were converted from original data using the ProteoWizard software (v3.0.8789). The parameters of alignment refer to the data from the published literature (34). In positive and negative ion modes, 10,288 and 12,565 precursor molecules were obtained, respectively. The metabolites were identified by mass spectra against reference spectra of human metabolome database,^1^ Metlin,^2^ massbank,^3^ LipidMaps,^4^ mzclound,^5^ and database built by BioNovoGene Co., LTD with a mass accuracy of 30 ppm. Excel was used for subsequent analysis. Then the intensity of the data was batch normalized.

### Data processing and analysis

SIMCA-P (v13.0) and R language ropls package were used for multivariate statistical analysis, including PCA and PLS-DA. The metabolites with VIP (Variable importance in the projection) values ≥1 and value of *p* ≤0.05 in the model were selected for Kyoto Encyclopedia of Genes and Genomes (KEGG) analysis. MetPA, mainly based on KEGG metabolic pathways, is a part of Metaboanalyst,^6^ which was selected for concentration and topology analysis of metabolic pathways to identify differential metabolic pathways. Pathway impact, value of p, and false discovery rate (FDR) value were obtained in the above step. The signal pathways with pathway impact >0.2, value of *p* <0.05, and FDR < 0.05 were screened. Metabolites with alterations with a 3-fold change compared to the control levels were selected. The obtained metabolites were analyzed by heat map. The metabolites with no numerical overlap in their concentrations in AD and control groups were screened as potential diagnose markers of AD.

### Statistical analysis

Data were presented as means ± standard deviation (SD). Statistical analyses were performed using SPSS 19.0 software. Significance test was conducted using unpaired two-tailed Student’s t-tests with value of *p* <0.05.

## Results

### Clinical data of the study population

The clinical data of all the recruited individuals are shown in Table 1. The ages of the two groups did not differ significantly. The number of individuals in each age group was displayed in the table.

**Table 1:** Clinical data of subjects.

Groups	Ctrl (*n* = 25)	AD (*n* = 26)	value of *p*
Ages (years)	39.48 ± 1.94	40.00 ± 1.70	0.84
20–29 years old (cases)	3	3	/
30–49 years old (cases)	16	18	/
50–59 years old (cases)	6	5	/
Gender	Male	Male	/

Data values of the ages were means ± standard error of the mean.

### Multivariate statistical analysis of metabolites

**Figure 2:**
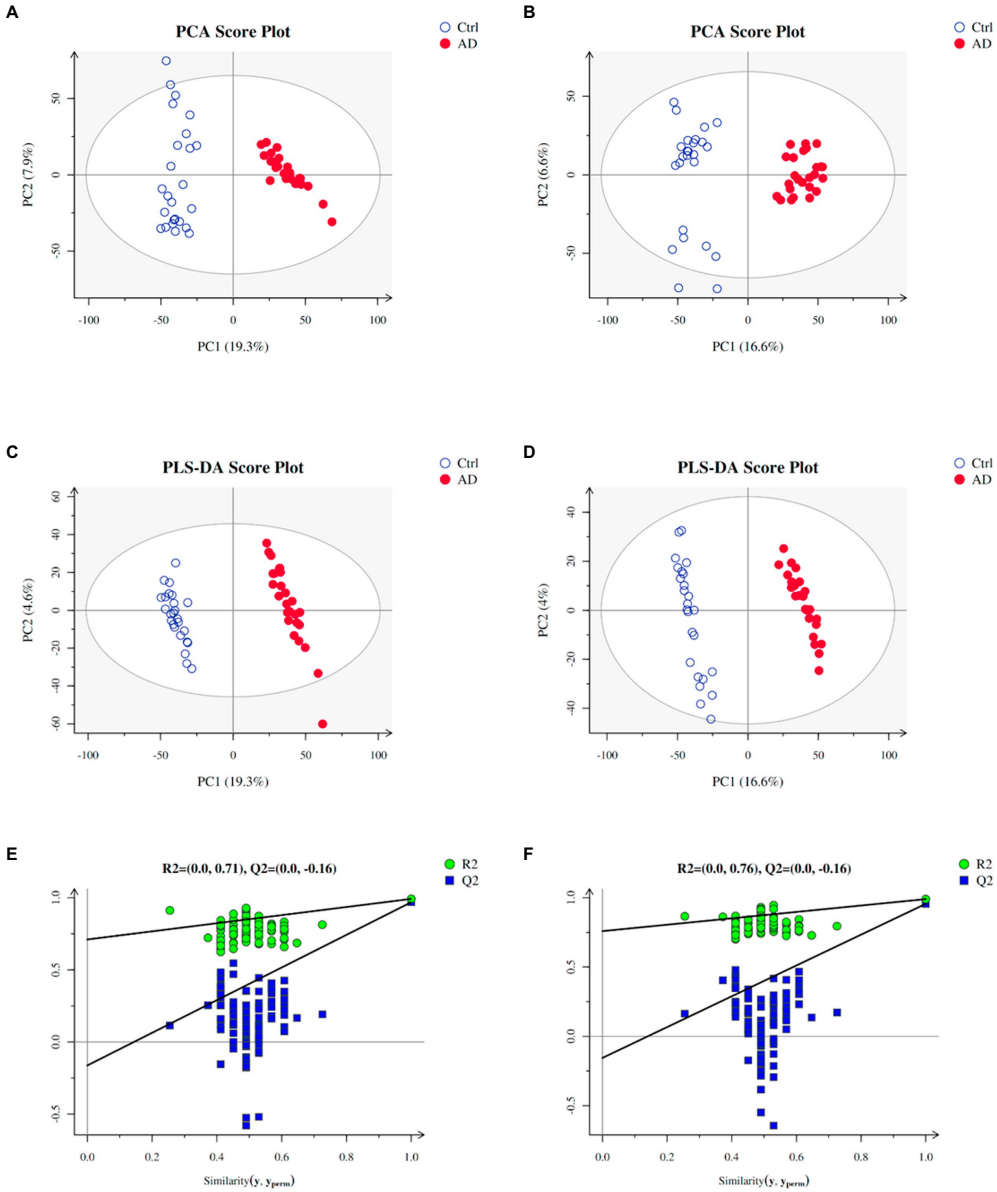
Plots of PCA and PLS-DA scores of the training samples. **(A)** PCA in positive ion mode for the sample. **(B)** PCA in negative ion mode for the sample. **(C)** Score plots of PLS-DA in positive ion mode. **(D)** Score plots of PLS-DA in negative ion mode. **(E)** A plot of PLS-DA permutation in positive ion mode. **(F)** A plot of PLS-DA permutation in negative ion mode. To determine that PLS-DA is not overfitting, one of two conditions needs to be met: (1) All blue Q2 points are lower from left to right than the original blue Q2 point, and (2) The regression line of point Q2 is less than or equal to 0 at the intersection of ordinates. Ctrl: control group; AD: alcohol dependence group.

Multivariate statistical analysis was used to process the metabolomics data. PCA score plot was used to depict the original state of the training sample data (The PCA diagram of all samples with QC samples was shown in Supplementary Figure S1). As depicted in Figures 2A,B, PCA can effectively separate the AD from control groups in the training set. PLS-DA was performed to screen different metabolites. Metabolites identified from positive and negative ion modes could be used to identify the differences between the control and the AD groups (Figures 2C,D), respectively. The permutation test showed no overfitting (Figures 2E,F). All our results illustrated reliable differentiation in metabolic changes between AD and control groups.

### Differential metabolite and pathway screening

**Figure 3:**
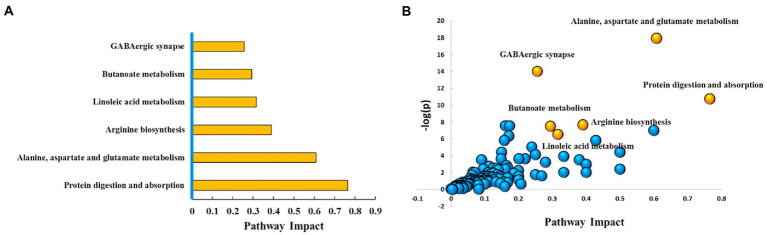
Top six metabolic pathways. **(A)** Histogram of top six metabolic pathways. **(B)** Bubble diagram of metabolic pathways. Each bubble represents a metabolic pathway. The top six pathways were highlighted in yellow color, and the rest remaining were in blue color. Based on the significant values of value of p, pathway impact score, and FDR (<0.05, >0.2, and < 0.05, respectively), the top six metabolic pathways were selected and shown by name. Ctrl: control group; AD: alcohol dependence group.

With VIP ≥ 1 and *p* ≤ 0.05 as the screening criteria, 154 differential metabolites were screened in the AD and the control groups, which were displayed in a heat map (Supplementary Figure S2). Further, we used the MetPA database (a part of the meta-analysis, mainly based on the KEGG metabolic pathway) to conduct pathway enrichment and topological analysis. The signal pathways with pathway impact >0.2, value of *p* <0.05, and FDR < 0.05 were screened. The top six selected signal pathways were protein digestion and absorption; alanine, aspartate, and glutamate metabolism; arginine biosynthesis; linoleic acid metabolism; butanoate metabolism; and GABAergic synapse. The information on the pathways was shown in Table 2. The histogram of the top six screened signal pathways was displayed in Figure 3A. The bubble diagram of all changed signal path diagrams was drawn in Figure 3B, wherein the top six screened pathways were highlighted with yellow.

**Table 2:** Six important pathways in alcohol dependence.

Pathway name	Pathway impact	-log(*p*)	value of *p*	FDR	Hits
Protein digestion and absorption	0.765	14.008	<0.001	<0.001	12
Alanine, aspartate and glutamate metabolism	0.608	17.935	<0.001	<0.001	11
Arginine biosynthesis	0.390	7.658	<0.001	0.017	6
Linoleic acid metabolism	0.316	6.534	0.001	0.037	6
Butanoate metabolism	0.294	7.511	0.001	0.017	8
GABAergic synapse	0.255	10.734	<0.001	0.001	5

### Selection of potential biomarkers

**Figure 4:**
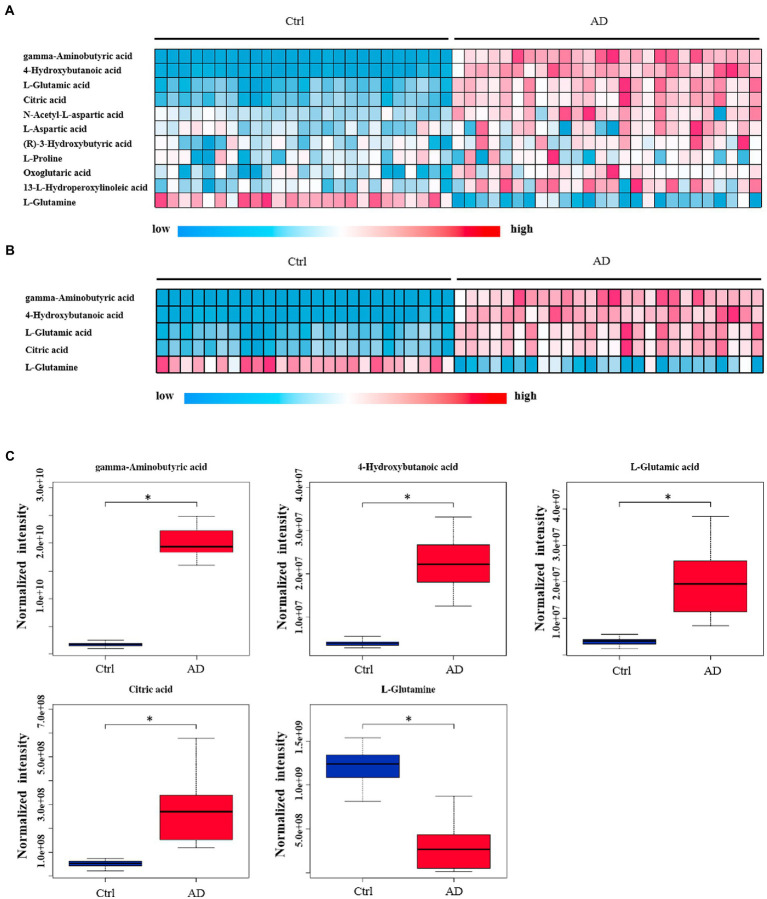
Heat map and box plot of important metabolites related to alcohol dependence. **(A)** Heat map of 11 metabolites screened from six signal pathways. **(B)** The metabolite heat map screened according to heat map A with a small difference within the group (with consistent color within the group). Columns represent samples, and rows represent metabolites. **(C)** Box plot of the selected five metabolites. Ctrl: control group; AD: alcohol dependence group. *p < 0.05.

The levels of 28 metabolites changed significantly in the top six selected signal pathways. The names of these metabolites, as well as the times they appear in the six metabolic pathways were shown in Table 3. 11 of these metabolites changed more than three-fold when compared to their respective levels in the control group. Gamma aminobutyric acid (GABA), 4-hydroxybutanoic acid, L-glutamic acid, citric acid, N-acetyl-L-aspartic acid, L-aspartic acid, (R)-3-hydroxybutyric acid, L-proline, oxoglutaric acid, 13-L-hydroperoxylinoleic acid, and L-glutamine were among the 11 metabolites identified (Table 4). Figure 4A depicts a heat map of these eleven metabolites. Metabolites with no numerical overlap in their concentrations in the AD and the control groups were screened out, as indicated by their heat map color. In the relative concentration data of L-glutamine, the control group and AD group had slightly higher and lower data, respectively. However, besides these two parameters, the other concentration data of the two groups did not overlap. Finally, five metabolites were screened out. Four of these five metabolites, including GABA, 4-hydroxybutanoic acid, L-glutamic acid, and citric acid, were significantly upregulated, and one, that is, L-glutamine, was significantly downregulated (Figure 4B). These five metabolites may be used as potential biomarkers. The box diagram of these five metabolites is displayed in Figure 4C.

**Table 3:** Times of occurrences in the six important signal pathways.

Times of occurrences in 6 important signal pathways	Name of metabolites
5	L-Glutamic acid; Oxoglutaric acid
3	Succinic acid; L-Aspartic acid; L-Glutamine; gamma-Aminobutyric acid
2	L-Arginine; L-Asparagine; Succinic acid semialdehyde; Argininosuccinic acid
1	L-Phenylalanine; L-Leucine; L-Histidine; L-Proline; Citric acid; Indole; L-Cystine; Diacetyl; 4-Hydroxybutanoic acid; (R)-3-Hydroxybutyric acid; N-Acetyl-L-aspartic acid; Linoleic acid; Piperidine; 13-L-Hydroperoxylinoleic acid; N-13S-hydroxyoctadecadienoic acid; 13-OxoODE; Alpha-dimorphecolic acid; 9(S)-HPODE

**Table 4:** Important metabolites screened for alcohol dependence.

UP or DOWN	Metabolite name	Fold change
**UP**	gamma-Aminobutyric acid	11.38
	4-Hydroxybutanoic acid	5.59
	L-Glutamic acid	5.40
	Citric acid	5.19
	N-Acetyl-L-aspartic acid	4.83
	L-Aspartic acid	4.83
	(R)-3-Hydroxybutyric acid	4.34
	L-Proline	4.16
	Oxoglutaric acid	4.15
	13-L-Hydroperoxylinoleic acid	3.20
**DOWN**	L-Glutamine	0.24

### Verification of the screened biomarkers

**Figure 5:**
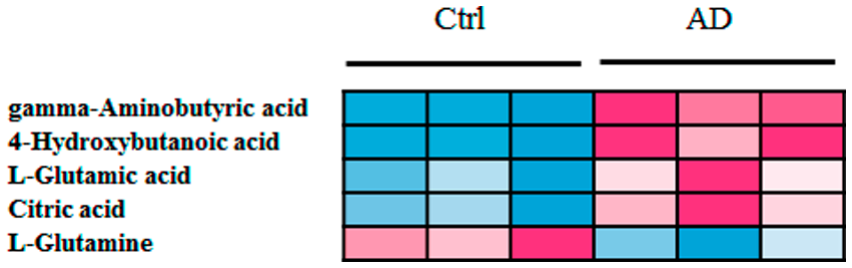
Heat map of screened metabolite markers in validation samples. Ctrl: control group; AD: alcohol dependence group.

Further, six samples were used to verify the screened biomarkers (i.e., GABA, 4-hydroxybutanoic acid, L-glutamic acid (L-Glutamate), citric acid, and L-glutamine) from the control and AD groups, respectively. The heat map of the potential biomarkers was shown in Figure 5. Our analysis results from the validation samples revealed no numerical overlap in the levels of the five screened metabolites between the AD and the control groups, which was consistent with our test results. Thus, these five screened metabolites were considered to be potential diagnostic markers for AD.

## Discussion

In this study, an untargeted UPLC-MS platform was used to analyze the serum metabolites of 29 unmedicated AD patients and 28 normal controls. 25 control and 26 AD samples were used in the training set, and the remaining samples used as the validation set. The PCA and PLS-DA scores were used for multivariate statistical analysis of the metabolites. Results of both PCA and PLS-DA analysis showed that the control and AD group could be distinguished effectively. We observed that the serum metabolites of AD patients differed significantly from those of the control population. This finding, in turn, demonstrated that alcohol, an energy-providing metabolite, significantly affects the serum metabolites of AD patients. MetPA database was then used to perform pathway enrichment (a part of the meta-analysis, mainly based on the KEGG metabolic pathway). Six important AD-related pathways were investigated. Twenty-eight metabolites were screened for changes in these six pathways. Eleven of these metabolites showed a ≥ 3-fold difference in levels between the AD and control groups. Furthermore, five metabolites with a stable difference between groups and a stable level in the groups were screened and considered as potential AD biomarkers using heat map analysis. In addition, we used six samples (three from each of the control and AD groups) to validate the screened biomarkers. GABA, 4-hydroxybutanoic acid, L-glutamic acid (L-Glutamate), citric acid, and L-glutamine were among the biomarkers studied.

A previous study revealed a significant increase and decrease in plasma glutamate and glutamine levels, respectively, in individuals with alcoholic liver diseases compared to those with non-alcoholic liver diseases (35). Consistent with this study, we found that the serum levels of L-glutamic acid and glutamine significantly increased and decreased, respectively, in AD patients. These findings indicated that alcohol consumption might be responsible for the altered plasma glutamate and glutamine levels. GABA, an inhibitory neurotransmitter in the central nervous system, is synthesized from glutamate through a process catalyzed by decarboxylase (36). In the current study, we found significant upregulation of the serum levels of GABA and 4-hydroxybutanoic acid. Previous studies have shown that alcohol promotes the release of GABA in the central amygdala (37). Although this may be a cause of increased GABA synthesis, it does not justify GABA upregulation in peripheral serum. We speculate that the higher serum GABA concentration is due to the effect of alcohol on body metabolism. GABA crosses the blood–brain barrier and affects the central nervous system, influencing the progression of AD. 4-Hydroxybutanoic acid,like GABA, has an ethanol-mimicking effect on the central nervous system (38). In the current study, we observed upregulation of serum citric acid levels. Very few studies have focused on the effects of alcohol on serum citric acid concentration. Notably, none of these studies held any reference value for the current study.

The small molecule metabolites in the serum mainly reflect the influence of drugs or the disease itself on body metabolism of the body. However, it is still unclear how these molecules can affect the nervous system. We propose that some of the small molecule metabolites can affect the central nervous system as neurotransmitters by passing through the blood–brain barrier, leading to the development of mental diseases such as AD.

The metabolic level in AD patients are differing for those of controls. A total of 28 significant alteration metabolites was screened and metabolic pathway analysis demonstrated that those altered metabolites were related to 6 biochemical pathways (i.e., protein digestion and absorption; alanine, aspartate, and glutamate metabolism; arginine biosynthesis; linoleic acid metabolism; butanoate metabolism; and GABAergic synapse). We discovered a significant change in 28 metabolites in these six pathways. Of these, the levels of 11 metabolites changes by at least 3-fold: GABA, 4-hydroxybutanoic acid, L-glutamic acid, citric acid, N-acetyl-L-aspartic acid, L-aspartic acid, (R)-3-hydroxybutyric acid, L-proline, oxoglutaric acid, 13-l-hydroperoxylinoleic acid, and L-glutamine. Among these, five metabolites with no numerical overlap in their concentrations between the AD and the control groups were GABA, 4-hydroxybutanoic acid, L-glutamic acid, citric acid, and L-glutamine. We postulate that these five metabolites could be used as potential diagnostic markers for AD.

## Data availability statement

The original contributions presented in the study are included in the article/Supplementary material , further inquiries can be directed to the corresponding authors.

## Ethics statement

The studies involving human participants were reviewed and approved by the Second Affiliated Hospital of Xinxiang Medical University. The patients/participants provided their written informed consent to participate in this study.

## Author contributions

YZ, YS, QM, CW, and RZ designed the study, conducted the literature search, and wrote the manuscript. QW, SG, TS, XG, SL, and GC contributed to the data analysis. QM, SG, and QW contributed to the collection of the samples. All authors contributed to the article and approved the submitted version.

## Funding

This work was supported by Science and Technology Research Project of Henan Province (Program no. 222102310330), Key Scientific Research Projects of Colleges and Universities in Henan Province (Program no. 22A320040), Science and Technology Project of Xinxiang (Program no. GG2020037), Open Project of Clinical Medical Research Center for Mental and Psychological Diseases of Henan Province (Program no. 2019-zxkfkt-002) and Medical Science and Technology Project of Henan Province (Program no. LHGJ20200529).

## Conflict of interest

The authors declare that the research was conducted in the absence of any commercial or financial relationships that could be construed as a potential conflict of interest.

## Publisher’s note

All claims expressed in this article are solely those of the authors and do not necessarily represent those of their affiliated organizations, or those of the publisher, the editors and the reviewers. Any product that may be evaluated in this article, or claim that may be made by its manufacturer, is not guaranteed or endorsed by the publisher.

## Footnotes

^1^ http://www.hmdb.ca

^2^ http://metlin.scripps.edu

^3^ http://www.massbank.jp/

^4^ http://www.lipidmaps.org

^5^ https://www.mzcloud.org

^6^ www.metaboanalyst.ca
