# Supplementary material for: Serum metabolomics analysis in patients with alcohol dependence
Source: Front Psychiatry. 2023 Apr 17;14:1151200. doi: 10.3389/fpsyt.2023.1151200 (PMC10150058; doi:10.3389/fpsyt.2023.1151200)
Supplement: Supplementary file 1 [file Data_Sheet_1.docx]

Supplementary Material

**Serum Metabolomics Analysis in Patients With Alcohol Dependence**

**Yanjie Zhang^a,b#^, Yajun Sun^a,c#^, Qin Miao^a,d#^, Shilong Guo^e^, Qi Wang^a,b^, Tianyuan Shi^a,b^, Xinsheng Guo^a,b^, Shuai Liu^a,b^, Guiding Cheng^a,b^, Chuansheng Wang ^a,b*^, Ruiling Zhang^a,b*^**

^a^ Henan Mental Hospital, The Second Affiliated Hospital of Xinxiang Medical University, Xinxiang, China

^b^ Henan Key Lab of Biological Psychiatry, Xinxiang Medical University, Xinxiang, China

^c^ Department of Scientific Research, The Second Affiliated Hospital of Xinxiang Medical University, Xinxiang, China

^d^Department of Addiction, The Second Affiliated Hospital of Xinxiang Medical University, Xinxiang, China

^e^Department of Oncology, The Third Affiliated Hospital of Xinxiang Medical University, Xinxiang, China

^#^These authors contribute equally to this work.

*** Correspondence:** Chuansheng Wang: chuansonwang@126.com

Ruiling Zhang: zhangruilingxxmu@126.com

## Supplementary Figure


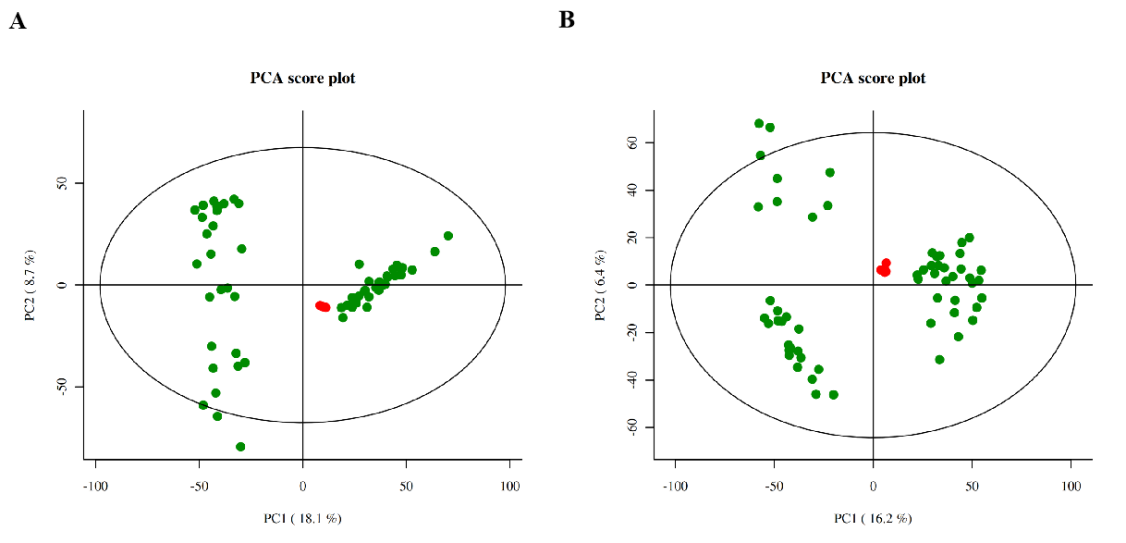


**Supplementary Figure 1.** **Plots of PCA scores of all samples.** (A) PCA in positive ion mode for all samples. (B) PCA in negative ion mode for all samples.


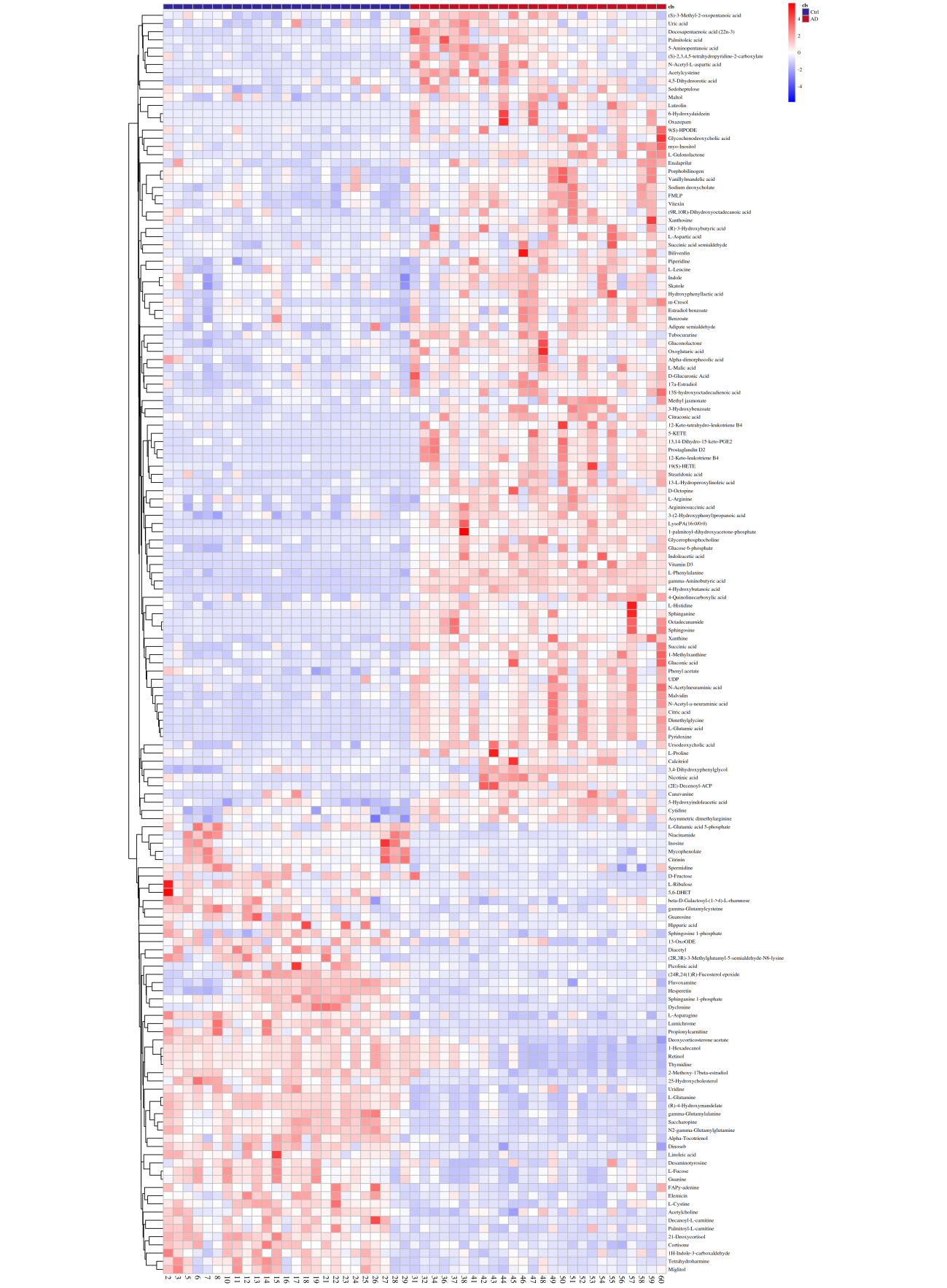


**Supplementary Figure 2.** Differential metabolite heat map. The columns represent samples, the rows represent metabolites, and the relative content of the metabolites is displayed by color. The heat map shows differential metabolites among AD, and control groups. Ctrl：control group；AD：alcohol dependence group
